# Preventive Pathways for Healthy Ageing: A Systematic Literature Review

**DOI:** 10.3390/geriatrics10010031

**Published:** 2025-02-18

**Authors:** Alice Masini, Niccolò Cherasco, Andrea Conti, Irlanda Pighini, Francesco Barone-Adesi, Massimiliano Panella

**Affiliations:** 1 Department of Translational Medicine, Università del Piemonte Orientale, 28100 Novara, Italy; alice.masini@uniupo.it (A.M.); 20051926@studenti.uniupo.it (N.C.); 10025977@studenti.uniupo.it (I.P.); francesco.baroneadesi@uniupo.it (F.B.-A.); massimiliano.panella@med.uniupo.it (M.P.); 2 Doctoral Program in Food, Health, and Longevity, Università del Piemonte Orientale, 28100 Novara, Italy

**Keywords:** healthy ageing, preventive pathways, lifestyle interventions, health promotion, active ageing, systematic literature review, elderly

## Abstract

**Background**: The world’s population is not only growing but also ageing, and healthcare systems should adapt to the needs of an ageing population. Until now, there has been no clear definition of a preventive pathway with the aim of improving lifestyles and promoting healthy and active ageing. The present systematic review aims to provide evidence to support the development of effective ways of delivering preventive pathways for healthy ageing. **Methods**: Several databases were searched, i.e., MEDLINE, COCHRANE, CINAHL, and PsycINFO, by using specific inclusion criteria, such as elderly population (i.e., subjects aged 65 years and older), preventive interventions for healthy ageing, studies with or without control groups, and effectiveness and methodological structure of the prevention pathway. The risk of bias was assessed by using the Joanna Briggs Institute and mixed methods appraisal tools. **Results**: A total of 9998 studies were identified after the removal of duplicates, and after screening title, abstracts, and full text, 14 studies were finally included. All the prevention pathways described are based on physical activity (PA) programmes, dietary interventions, and cognitive and mental health. The professional figures involved in the pathways were experts in prevention and health promotion, like family and community nurses, kinesiologists, and experts in stress management. The majority of the preventive pathways were implemented in primary care and community settings. **Conclusions**: Our systematic review provides evidence for developing an effective preventive healthy ageing pathway through tailored PA, diet, and cognitive health interventions. This co-designed approach should involve a multidisciplinary expert team and be implemented in primary care and community settings to improve psycho-physical health and longevity.

## 1. Introduction

Health promotion and healthy ageing have become imperatives today, reflecting a major shift in our society towards an emphasis on healthy lifestyles and prevention. In recent years, and in response to this need, the World Health Organization (WHO) announced that the concept of health promotion is the most effective way to promote human health [1]. In fact, the world’s population is not only growing; it is also ageing. This phenomenon is known as the “third demographic transition”, and it is estimated that by 2050, there will be an increase in the number of people aged 65 and over, i.e., the elderly [2]. The European Commission estimates that by 2060, the proportion of European citizens aged over 65 will increase from 18% to 28% and the proportion aged over 80 will increase from 5% to 12% (European Commission, 2015). Ageing is a process that although it is intrinsic to human nature, is influenced by a complex interaction among biological, cultural, community, and environmental aspects. The WHO defines active ageing as “the process of optimising opportunities for health, participation, and security in order to enhance quality of life as people age” for “helping people stay in charge of their own lives for as long as possible as they age and, where possible, to contribute to the economy and society” [3].

The demographic transition towards an aging population presents multifaceted consequences that not only affect individual health trajectories but also pose substantial socioeconomic challenges and put strain on healthcare delivery systems. To address these challenges, more than 190 countries endorsed the Global Strategy and Action Plan on Ageing and Health (2016–2030). This plan aims to promote healthy ageing, defined as “the process of developing and maintaining the functional ability (i.e., people’s capabilities of being and doing what they have reason to value) that enables well-being in older age” [4]. Therefore, the goal of healthy ageing should be primarily focused on improving physical, psychological, and mental health in the population aged 60 and over [5].

In consideration of the ageing process, we need to take into account that frailty-related conditions such as chronic diseases are rising. This trend creates new health needs for the population and requires a shift from an acute care model to a coordinated and comprehensive continuum of care. Healthcare systems need to adapt to this change and reorganise themselves, as current models of care are inadequate to meet the health needs of a rapidly ageing population [6].

The WHO urges member states to base their quality improvement policies on the entire continuum of care, taking into account the criteria of effectiveness, safety, equity, efficiency, integration of care, and timeliness [7]. To this end, a continuum of care should serve to reduce medical costs while providing optimal patient-centred care [8]. A care pathway is a complex intervention for the mutual decision making and organisation of care processes for a well-defined group of patients during a well-defined period [9]. The majority of care pathways are tailored for a specific disease. To date, no clear definition of a preventive pathway aimed to improve active and healthy ageing exists. According to the WHO, living arrangements including social support, social wealth, and background factors (e.g., age, gender, marital status, employment status, educational background, income, and size of the family) are considered influential in active and healthy ageing [10].

In order to improve both longevity and quality of life in older adults, the lifestyle approach is one of the most promising strategies for enhancing longevity and quality of life in older adults [5]. Lifestyle factors are key targets for behaviour change interventions focused on promoting healthy ageing with PA, nutrition, and cognitive function [11].

Therefore, our study is aimed at supporting the development of preventive pathways for healthy ageing through a systematic review of the most recent scientific evidence.

## 2. Materials and Methods

### 2.1. Search Strategy

This systematic review was conducted in accordance with the PRISMA guidelines [12]. The study protocol is available in the International Prospective Register of Systematic Reviews—PROSPERO (ID: CRD42023431045). The search strings were developed considering the following PICOS (Patients, Interventions, Comparators, Outcomes, and Study design): (P) older adults, namely, subjects aged 65 years or over; (I) preventive pathways or preventive programs for healthy ageing; (C) both studies with and without control groups were included; (O) efficacy and methodological structure of the existing preventive pathway or preventive programs; (S) primary research based on original data. Detailed PICOS criteria are available in Table 1.

### 2.2. Selection Criteria

The literature search was conducted in June 2023 on MEDLINE (PubMed), Cochrane Central Register of Controlled Trials, Cumulative Index to Nursing and Allied Health Literature (CINAHL), Psychological Information Database (PsycINFO) (EBSCO), and Scopus. We searched electronic databases, with a 10-year publication date limit, because we were interested in recent approaches. Search strategies (strings adapted when necessary to fit the specific search requirements of each database) used the following keywords combined with Boolean Expressions (i.e., queries using logical operators “AND”, “OR” and “NOT”, between the main terms): ((Pathway* OR Path OR Approach) AND (“Preventive Health Services”[Majr] OR Care Preventive Health OR Health Care Preventive OR Preventive Care OR Preventive Health OR Health Preventive OR Preventive Health Programs OR Health Program Preventive OR Health Programs Preventive OR Preventive Health Program OR Program Preventive Health OR Programs Preventive Health OR Preventive Programs OR Preventive Program OR Program Preventive OR Programs Preventive) AND (“Aged”[Mesh] OR elderly) AND (“Methods”[Mesh] OR methodology OR barrier* OR facilitator* OR Design OR Model* OR Modeling OR Model* composition OR Current organization) NOT (laboratory)). Filters applied: Full text; published in the last 10 years; Humans; English; Aged: 65+ years, 80, and over (80+ years). The inclusion criteria were as follows: (1) language: articles written in English; (2) study design: both experimental and observational studies with original primary data; (3) population of interest: older adults and the elderly with mean age >65 years with or with no chronic conditions; (4) intervention: preventive pathways or preventive programs for healthy ageing; (5) outcome measurement: efficacy, and methodological structure and characteristics of the existing preventive pathway or preventive programs; (6) comparison: not relevant, both studies with or without control groups were included. The exclusion criteria were as follows: (1) articles not pertinent to the research topic; (2) populations of a different age (adult mean age <65 years); (3) studies with interventions also including other topics; (4) study protocols or other papers without original data. Moreover, we conducted a grey literature search of other papers, using hand searches of key conference proceedings, journals, professional organisations’ websites, and guideline clearing houses. Finally, with a snowball technique, we examined references cited in the primary papers to identify additional eligible papers.

### 2.3. Data Extraction

After the removal of duplicates performed by adopting Zotero (Roy Rosenzweig Center for History and New Media; www.zotero.org/; (accessed on 24 October 2024)), title and abstract screening was performed with Microsoft Excel (Microsoft Corp, Redmond, PA, USA). This was conducted by a total of three researchers. Specifically, two researchers (N.C. and I.P.) screened the records, while the third one (A.C.) was consulted in case of disagreement. The research team contacted the study authors in the case where more information was deemed necessary. The same approach was adopted for full-text screening and data extraction. The retrieved information was inserted in a Google Sheet database, which included the following fields: name of the first author, publication year, country, study design, population details (including age and gender), intervention description (including setting, duration, and measured outcomes), and results. Data extraction followed the methods provided by the Cochrane Reviewers’ Handbook [13]. The data extraction phase was performed manually, and the content of each included paper was thematically analysed.

### 2.4. Risk of Bias Assessment

To assess the risk of bias for the potentially included studies, two reviewers (A.C. and I.P.) independently and blindly applied the Joanna Briggs Institute (JBI) critical appraisal instruments [14,15,16] and the mixed methods appraisal tool (MMAT) [17]. The tiebreaker (A.M.) was consulted in case of conflict. The risk of bias evaluation was made based on the primary outcome of interest: methodological structure and characteristics of the existing preventive pathway or preventive program, according to the PRISMA guidelines [12]. The JBI checklist for randomized controlled trial (RCT) studies [14] comprises thirteen items designed to evaluate different methodological aspects, including the randomisation and the allocation concealment process, the blindness of participants and researchers, and the statistical analysis used. Items four and five (“Were participants blind to treatment assignment?”; “Were those delivering the treatment blind to treatment assignment”) were not considered, due to the nature of the included studies which investigated lifestyle intervention. The JBI checklist for quasi-experimental (QES) studies [15] comprises nine items designed to evaluate different methodological aspects of this kind of studies, including the methods, the outcomes, the characteristics of the participants, the description of the treatment, control, follow-up, and the statistical analysis used. The JBI checklist for qualitative studies (QSs) [16] comprises ten items designed to evaluate different methodological aspects of this kind of studies, including five items for assessing research methodology congruity (i.e., stated philosophical perspective, research question or objectives, methods used to collect data, and representation and analysis of data). Each checklist presents different possible answers: “yes”, “no”, “unclear”, or “not applicable”. The MMAT scale [17] is designed for the appraisal stage of mixed methods studies (MMSs) comprising two parts, where the first part has questions related to qualitative design and the second part for quantitative design. The criteria to generate the overall score were decided and approved by all the authors. In detail, the overall score was assigned as follows: (i) “High quality” if all the criteria were met. (ii) “Medium quality” if at least one domain was unclear. (iii) “Low quality” if at least one domain was not met.

## 3. Results

### 3.1. General Characteristics

**Figure 1:**
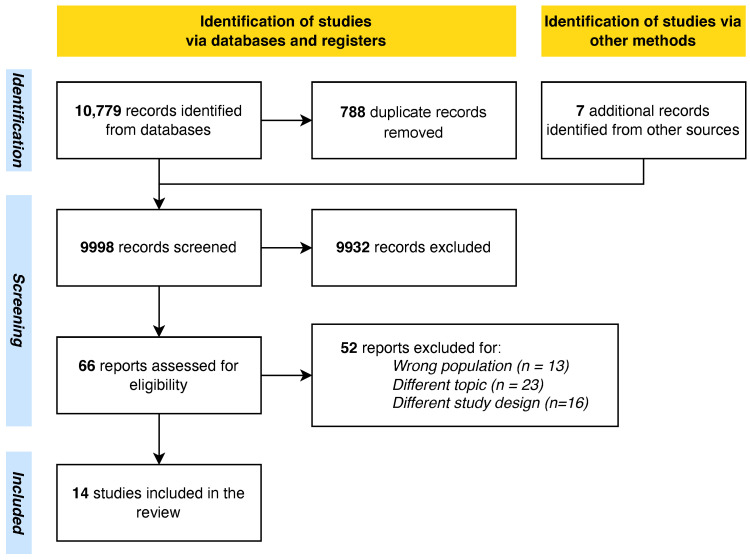
PRISMA flowchart.

A total of 9998 unique records were retrieved after duplicate removal. A total of 261 articles were passed in the abstract screening phase. Finally, 14 studies fully meeting the eligibility criteria were included in this systematic review (Figure 1). The characteristics of the studies are shown in Table 2.

The geographic origins of the study were as follows: United Kingdom (n = 1) [19], United States (n = 1) [27], The Netherlands (n = 3) [21,22,29], Canada (n = 1) [31], Germany (n = 2) [18,26], Japan (n = 1) [28], China (n = 3) [24,25,30], Iran (n = 1) [20], and Australia (n = 1) [23]. The studies were published between 2015 and 2023. The study designs were RCT = 7 [18,19,20,25,28,29,30], quasi-experimental = 3 [21,24,27], qualitative = 2 [23,26], and mixed methods = 2 [22,31]. The age of the population ranged from 60 to 85 years old, while the sample size varied from 34 to 986 participants. The study duration ranged from 12 weeks to 12 months.

### 3.2. Risk of Bias Results

Table 3 summarises the quality of the included studies assessed with different scales. The complete quality appraisal is available in the Supplementary Materials (Tables S1–S4).

With regards to the randomised control trials, both Davodi et al. [20] and Beyer et al. [18] were scored as being of “low quality” due to the absence of a clear randomisation process, blindness of assessors, and follow-up data. A medium-quality result was obtained by three studies [19,25,29]. Finally, two studies [28,30] obtained a high level of quality due to the correct explanation for all domains. As concerns the quasi-experimental studies, only one study [21] obtained a high level due to the correct answers given in each evaluation field, while Schwingel et al. [27] was assessed as being of low quality due to the absence of a control group and follow-up, and one study [24] was rated as being of medium quality for the unclear follow-up description [23,26]. The qualitative study performed by Green et al. [23] obtained a medium-quality rating due to the lack of clarity regarding the researcher’s cultural or theoretical positioning, while the other qualitative study [26] was scored as being of high quality. Finally, the mixed methods studies conducted by Franse et al. [22] and by Yusupov et al. [31] obtained medium quality due to the unclear aspect of the quantitative components of the study, such as the completed outcome data and the representative sample size.

### 3.3. Preventive Pathway Characteristics

The 14 included studies reported detailed information related to the preventive pathway interventions implemented during their project. The complete data extraction is shown in Appendix A. The preventive pathways examined in the included studies were based on various specific lifestyle interventions aimed at healthy aging.

#### 3.3.1. Physical Activity Component

The majority of the preventive pathways included a fundamental component of PA [18,19,20,21,22,23,24,25,27,28,29]. The main difference among the studies was self-managed PA [19,24,27,28,30] compared with other studies in which the activities were totally supervised by professionals or researchers [18,20,21,22,25,29]. The PA program was delivered in both a supervised manner and a non-supervised manner, like in the “Moving well” component of the “Being your Best” preventive pathway conducted by Green et al., in which multicomponent PA was delivered through group fitness activities in the community and educational booklets for home-based intervention [23]. The supervised PA programs were tailored for older adults and seniors and designed to suit the needs and the physical abilities of this age group. They are crucial to maintaining or improving mobility, muscle strength, and balance, which are key aspects of reducing the risk of falls. Particularly, refs. [22,27] included a specific PA program to reduce falls in the elderly. Self-managed PA varied from studies in which a booklet with exercises was given to the participants, who managed on their own all the activities [19,24], to other studies where a counselling process was implemented [27,30] in order to monitor adherence to the intervention, and in other studies, participants were invited to join extra PA group activities in the territory. In the study conducted by [19], each participant could decide the amount of time to devote to specific activities in order to be more realistic in developing an approach that could potentially be disseminated at a population level [19]. Moreover, the “Agewell” preventive pathway performed by [19] compared two experimental groups: one group with mentoring during the activities and the other with just goal setting. The most important exercise components of PA intervention, both in supervised and non-supervised activities, included in the pathway were balance exercises, strength exercises, aerobic activities (i.e., walking, swimming, or cycling), and flexibility. These exercises are often combined into a comprehensive program that also takes into consideration individuals’ pre-existing medical conditions, personal preferences, and health goals. All the 12 studies [18,19,20,21,22,23,24,25,27,28,29,30] which included PA programs in the preventive pathway showed important effects of increasing PA levels, preventing injuries and illnesses, and promoting a sense of overall well-being and self-esteem, encouraging older adults to lead an active and fulfilling lifestyle. The results of the included studies showed an improvement in physical health outcomes assessed with different objectives, such as actigraphs, fitness tests (senior fitness test, physical performance battery, and gait speed), steps counts, Falls Efficacy Scale International (FES-I) and self-reported measurements like the PA Scale for the Elderly PASE, sedentary behaviours (hours per day), regular exercise (number of sessions per week), body composition (i.e., BMI, body fat percentage), blood sample (i.e., cholesterol levels), cardiovascular risk, and interviews.

#### 3.3.2. Dietary Component

A total of nine studies [19,20,23,24,25,27,28,29,30] included a dietary intervention in the preventive pathway. The characteristics of this dietary component varied from nutritional general advice to booklets with diet recommendations or small group lessons to ensure that participants received a balanced diet that contributes to the prevention of chronic diseases and the maintenance of optimal health [19,20,23,24,25,27,28,30]. Only in the “SLIMMER” preventive pathway, a personalised program (i.e., a specific diet routine) was implemented for all the participants [29]. The effectiveness of the dietary components of the preventive pathway described were assessed by using specific questionnaires for adherence to the Mediterranean diet, like Medas, Kidmed, and food frequency questionnaires, which underlined a significant effect of adopting a more healthy diet and increasing adherence to the Mediterranean diet.

#### 3.3.3. Cognitive Training and Mental Health

Aspects of cognitive training for healthy ageing were included in nine studies [18,19,20,23,24,25,27,28,31]. The type of activities implemented varied from memory training, social gatherings, and group therapy to support mental health, better deal with the ageing process, and prevent isolation, which can negatively impact overall health. Moreover, two studies [24,28] implemented fundamental training related to digital knowledge, the internet, and social networks to obtain a positive effect on digital health literacy. Cognitive and executive improvements were obtained in the majority of the studies [18,19,20,23,24,25,27,28,31]. Several instruments were implemented in order to assess cognitive and executive functions (i.e., the Montreal Cognitive Assessment (MoCA), the Florida Cognitive Activities Scale (FCAS), and the California Verbal Learning Test (CVLT)). Moreover, depressive symptoms and psychological well-being were monitored (i.e., the German Version of the Center of Epidemiological Studies Depression Scale (CED-D 10), the Geriatric Depression Scale (GDS), and the General Self-Efficacy Scale (GSE)). Yusopov et al. is the only included study who based the preventive pathway for healthy ageing entirely on an online cognitive training programme called “memory and ageing”. The researchers involved the elderly in a previous qualitative phase in order to have their feedback to develop the app [31]. Among the cognitive and mental health outcomes, three studies were focused specifically on stress [20,24,27], proposing coping strategies and counselling activities in order to improve stress management during ageing.

#### 3.3.4. Other Characteristics

Among the included studies, other interventions were included in the preventive pathway. Refs. [24,30] incorporated the fundamental component of financial security in their preventive pathway in order to improve the economic skills of the elderly. The two studies conducted by Franse et al. [21,22] included a fundamental component related to polypharmacy and loneliness in the “UHCE” preventive pathway. The “UHCE” program was designed according to a bottom-up approach starting from a qualitative design. Refs. [24,25] included important chronic disease prevention and management support and training in the preventive pathway.

Finally, ref. [26] created the "Reaching the elderly" preventive pathway totally based on home visits to address the specific needs of older adults in their home environment. This pathway was designed according to feedback obtained from semi-structured interviews and focus groups. Lastly, ref. [20] was the only one that included spiritual aspects in the preventive pathway as fundamental to the ageing process of the Iranian population.

### 3.4. Professional Figures and Settings of Preventive Pathways

Several professional figures managed the preventive pathway components. First of all medical doctors, general practitioners, physicians, and experts in geriatrics coordinated many preventive pathways, in particular the enrolment and the evaluation phase to start the program [20,21,22,23,24,25,29,30]. Nurses were fundamental to the assessment phase and counselling activities in different programs [21,22,23,25,29]. The prevention pathways involved new professionals—kinesiologists and exercise trainers/experts—who collaborated with physiotherapists to manage exercise and PA components [18,20,21,22,24,25,28,29].

The cognitive and mental health components were managed by a psychologist or expert in mental health only in five studies [18,20,21,22,24]. Finally, the component relating to a healthy diet was managed by nutrition experts in only three studies [25,26,29]. Other fundamental professionals were involved in the care pathways, like social workers, social scientists, pharmacists, volunteers, e-learning designers, and experts in family relations and in social protection [25,26,29]. The only two studies who never mentioned in detail the professional figures involved in the program and just mentioned “research teams” were Clare et al. [19] and Yusopov et al. [31]. The study conducted by Schwingel A. et al. [27] involved a specific figure in the pathway and called them “Promotoras”. The researchers recruited and trained older women in the community for about 18 h throughout nine training modules that lasted about 2 h each. In several studies, religion was used as a means of facilitating community participation in prevention. For example, in the study by Davodi et al. [20], the inclusion of spiritual aspects was considered fundamental to the ageing process of the Iranian population, while in the study by Schwingel [27], the church and priests were the first point of contact with the Latino community and the starting point for implementing the programme. As concerns the setting of the preventive pathways, in the study conducted by [26], “Reaching the elderly” was the only programme based on home visits. The majority of the preventive pathways were performed in primary healthcare settings [21,22,25,29], community dwellings [18,23,27,31], or community centres [19,24,28,30]. Finally, ref. [20] conducted their “Active ageing” preventive pathway in a health and treatment centre.

## 4. Discussion

### 4.1. Main Results: Preventive Pathway Characteristics

**Figure 2:**
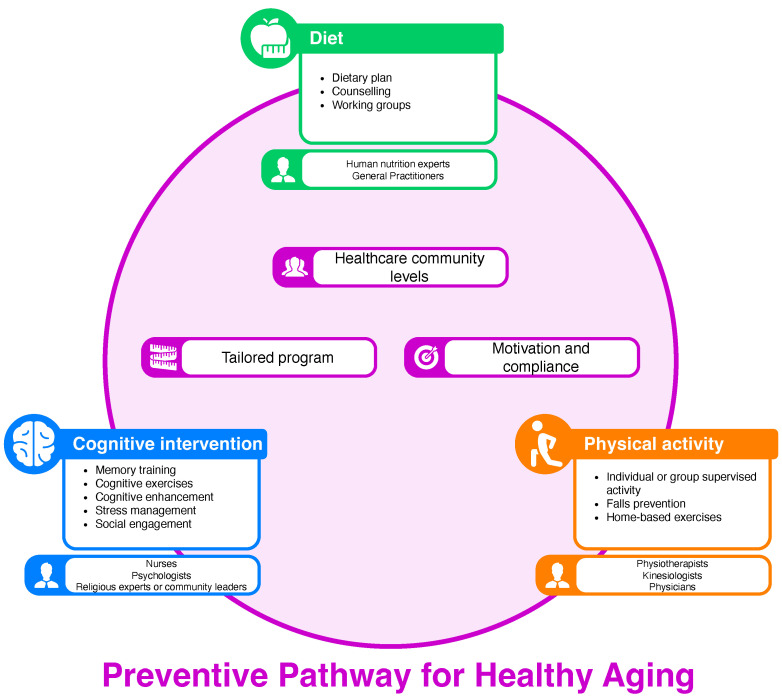
Preventive pathway characteristics.

The present is the first systematic review summarising 14 recent studies related to preventive pathways for healthy ageing. Overall, the present systematic review provides evidence to develop an effective preventive pathway for healthy ageing for older adults and the elderly. Figure 2 summarises the main characteristics of a preventive pathway for healthy aging.

Starting from the characteristics of the preventive pathways, the majority of the included studies focused the preventive program on lifestyle interventions, particularly PA, nutrition, and cognitive and mental health. These characteristics are in line with the aim of prevention and health promotion for healthy ageing proposed by the WHO [32] and the recent literature, which proves how lifestyle intervention represents a best buy in public health [5]. A growing body of literature suggests that the improvement in the health of older adults will not be achieved through a healthy lifestyle [33,34,35]. PA, nutrition, cognitive enhancement, stress management, and social engagement represent the main pillars of lifestyle medicine [36,37]. Moreover, the most effective preventive pathways for healthy ageing included in our systematic review pay attention to the social and psychological needs of the elderly [18,19,20,21,22,23,25,26,27,28,29,30]. Considering that older adults and the elderly have complex needs, the healthcare system should be adapted to meet their requirements, especially since the demand will increase as the population ages. These results are in line with the recent guidelines about healthy ageing priorities proposed by the WHO [38]. In fact, most studies have not only created a tailored program aimed at improving lifestyles but have also tried to take into consideration the motivation and compliance of the subjects involved. The personalisation of the interventions to meet these needs is essential and unique to older adults, highlighting how well-designed interventions can actively improve ageing, increase involvement in healthy activities, and above all, mitigate risk factors for chronic diseases [27]. In the majority of the included studies, the implemented design takes into account theoretical models of changed behaviour [39] or different behavioural models, like the socioecological model [40], on which to base programs in order to improve adherence and compliance. Precisely for this reason, several preventive programs were based on PA and cognitive intervention led by professionals but also tasks and activities that can be handled independently by the individual to facilitate adherence to and the management of the pathway without excessive limitations [19]. It is in this scenario that making the pathways accessible online represents an innovative aspect to be considered, like in the study performed by Yusopov [31], which takes into consideration the use of technology to spread these good healthy ageing practices. In addition, the results of our systematic review underline how the participation of older adults and the elderly in the creation of a preventive pathway is fundamental to its effectiveness and sustainability over time. In fact, among the included studies, the majority have envisaged a co-design phase of the intervention or a qualitative assessment [19,21,23,25,26,27,29,30,31], thus adopting a qualitative study design in which the interested stakeholders were involved to best co-design the pathways based on the needs that emerged. Incorporating the perspectives of the elderly and care providers is essential to establishing principles that make community health centres more age-friendly and better equipped to meet the needs of patients and the communities they serve [41].

### 4.2. Professional Figures and Settings

The adoption of these preventive pathways, which have demonstrated significant effectiveness in terms of improving psycho-physical well-being and quality of life, also presents numerous challenges, including cultural and linguistic barriers, which can undermine effectiveness in the elderly community. This trend was observed in studies conducted in low–middle-income countries [20,27]. For example, in the study by [27], Latino participants expressed mistrust towards research, society, and the healthcare system. This mistrust can negatively influence participation in health promotion programs. Promoting healthy lifestyles among these populations requires a culturally sensitive approach. This is the reason why the intercession of the so-called “promotoras” (community leaders) was fundamental and proved effective, as they spoke the local language and understood the culture of the participants. The “promotoras” receive specific training to transmit health information in an accessible and understandable manner. Moreover, in low–middle-income countries, religious experts were involved in the preventive pathway as one of the main contact points in order to improve adherence to the program. Furthermore, the present systematic review emphasises the growing necessity of involving new professional figures in preventive pathways. This includes not only medical and nursing staff, especially the emerging role of community and family nurses [42] in dealing with chronic conditions, but also experts in prevention and health promotion who collaborated together. Notably, professionals such as kinesiologists are becoming increasingly significant as physical exercise specialists, particularly in programs conducted outside traditional healthcare settings. For older adults, it is important to begin any new exercise program under the guidance of qualified professionals, such as physiotherapists and kinesiologists or graduates in sports science specialising in training older adults, to ensure that exercises are performed safely and with the right intensity even when self-managed. As regards the management of the healthy diet component, a different situation emerges. In most preventive programs, this component is managed by various professional figures who are not specifically trained on the topic. Only the studies conducted by Van Dongen al., Patzel et al., and Lee et al. enrolled an expert in healthy diet and nutrition in the multidisciplinary team [25,26,29]. Generally, the results of this systematic review underline the importance of continuing organisational support, clinical champions who communicate regularly with healthcare professionals, dedicated staffing, and ongoing data collection to easily and timely implement appropriate interventions. As regards the implementation setting, it is well recognised that addressing the growing burden of chronic diseases necessitates opportunities for health promotion and disease prevention within the community, as well as effective disease management within healthcare services. Several chronic diseases and related disabilities that could affect a person’s life span, along with their economic and human costs, can be prevented. However, prevention means that action needs to be taken before the start of the disease or before the disease takes hold, and that means taking a life course approach to active and healthy ageing [41]. Primary healthcare centre settings and community dwellings represents the most frequent environments in which the majority of preventive healthcare and screening for early disease detection and management take place. These primary healthcare centres, to which people can self-refer, also provide the bulk of ongoing management and care. It is estimated that 80% of front-line healthcare is provided at the community level, with primary healthcare serving as the foundation of the healthcare system [41]. Integrated care aims to transition from inpatient to ambulatory and outpatient care, emphasising home-based interventions, community engagement, and a well-coordinated referral system, as described in the majority of the included studies. This integration should happen at the healthcare organisation and community levels, as well as within policies, economic mechanisms, and shared governance structures. Furthermore, an integrated care system involves diverse professional figures operating in a variety of settings [43].

### 4.3. Limitations

This systematic review presents some limitations. First of all, we only searched for articles related to preventive pathways for the elderly aged over 65 years old from the last 10 years, since we were interested in recent approaches. The recent literature suggests that the healthy ageing process and new interventions should start even before then, during childhood and adolescence. Even if this systematic review followed the current methodology [12], it is worth mentioning that the search string terms might not be completely exhaustive and could have led us to miss some studies. Moreover, the included studies presented several heterogeneity. First of all, some studies have relatively small samples [19,23], which may limit the generality of the results. A larger sample could provide more robust and representative data. The variability in the methods and study designs produces some difficulties in the direct comparison of the results. Finally, the insufficient long-time follow-up limits the understanding of the lasting effects of interventions. It is essential to include prolonged follow-ups to evaluate the sustainability of the benefits of the interventions.

## 5. Conclusions

The present systematic review provides evidence to develop an effective preventive pathway for healthy ageing characterised by tailored co-design PA, healthy diet, and cognitive health components considering the levels of self-perceived motivation to change lifestyle. The future so-called “preventive pathway for healthy ageing” should present a multidisciplinary task force with several professionals and experts in health promotion and prevention. Preventive pathways should be implemented in primary care and community settings involving local community leaders in the development phase in order to be effective in improving psycho-physical health and increase longevity.

## Figures and Tables

**Table 1:** PICOS eligibility criteria.

Parameter	Inclusion Criteria	Exclusion Criteria
Population	Older adult/elderly * (i.e., subject aged 65 years and older) with or without chronic conditions	Adult mean age lower than <65 years
Intervention	Preventive pathways or preventive programs for healthy aging	Critical care pathway
Comparator	N/A	N/A
Outcome	Methodological structure and characteristics of the existing preventive pathway or preventive programs	
Study design	Experimental (i.e., RCTs, quasi-experimental studies, and pilot studies), observational study (i.e., cohort studies, longitudinal studies, and qualitative studies), or document papers describing the methodology of quasi-experimental or observational studies. Studies with full-text written in English.	Systematic reviews, meta analyses, or other papers without original data (i.e., reviews, letters to editors, editorials, book chapters, and conference abstracts).

* The terms “older adult” and “elderly” are used as synonyms in this review.

**Table 2:** Characteristics of the included studies.

Study and Year	Country	Study Design	Subjects (n)	Age (Mean)	Gender (Female, %)	Setting
Beyer et al., 2019 [18]	Germany	RCT	Total: 84; CG: 38; EG: 46.	Total: 76.8 ± 5.29.	65.2	Community dwelling
Clare L. et al., 2015 [19]	United Kingdom	RCT	Total: 75; IG: 27; GSG: 24; GSM: 24.	Total: 68.21; IG: 70.22 ± 7.77; GSG: 67.50 ± 7.66; GSM: 68.21 ± 7.92.	Total: 86.7; IG: 85.2; GSG: 95; GSM: 79.2.	Community centre
Davodi et al., 2023 [20]	Iran	RCT	Total: 60; CG: 30; EG: 30.	CG: 78.16 ± 7.07; EG: 66.5 ± 5.1	CG: 56.7; EG: 46.6.	Health and treatment centre
Franse et al., 2018 [21]	The Netherlands	Quasi-experimental	Total: 1844; CG 858; EG: 986.	Total: 79.5 ± 5.6; CG: 79.7 ± 5.5EG: 79.3 ± 5.7.	Total: 60.8; CG: 61.4; EG: 60.3.	Primary care and community settings in five European cities
Franse al. 2019 [22]	Germany	Mixed methods	Total: 986; FCP: 278; PCP: 130; LCP: 223; MCP: 94.	Total: >75 years.	EG1: 70.1; EG2: 50.8; EG3: 71.3; EG4: 62.8.	Primary care and community settings
Green et al. 2021 al. [23]	Australia	Qualitative	Total: 40; consumers: 23; professionals: 17.	Consumers: 69.3 ± 15.	Consumers: 65.	Community dwelling
Hsu et al., 2018 [24]	Taiwan	Quasi-experimental	Total: 123; CG: 43; EG person to person: 61; EG person to digital: 54.	CG: 78.16 ± 7.07; EG person to person: 77.25 ± 8.27; EG person to digital: 76.57 ± 7.01.	CG: 71.9; EG: 74.1.	Community care centres
Lee et al., 2021 [25]	Taiwan	RCT	Total: 398; EG: 199; CG: 199.	EG: 73.2 ± 6.1; CG: 73.1 ± 6.5.	EG: 62.0; CG: 58.0.	Primary healthcare setting
Patzelt C. et al., 2016 [26]	Germany	Qualitative	Total: 42.	Total: 65 and older.	Total: 52.4.	Community settings
Schwingel A. et al., 2017 [27]	USA	Quasi-experimental	Total: 34.	Total: 64 ± 8.	N/A	Community dwelling
Uemura et al. 2020 [28]	Japan	RCT	Total: 60; EG: 30; CG: 30.	EG: 74.0 ± 4.9; CG: 73.9 ± 4.4.	EG: 66.6; CG: 66.6.	Rural community
van Dongen et al., 2016 [29]	The Netherlands	RCT	Total: 316; EG: 155; CG: 161.	EG: 60.7 ± 6.4; CG: 61.0 ± 6.5.	EG: 47.7; CG: 50.3.	Dutch primary healthcare
Wong et.al, 2022 [30]	China	RCT	Total: 92; EG: 46; CG: 46.	Total: 75.9 ± 7.8; EG: 73.7 ± 6.9; CG: 78.2 ± 8.1.	Total: 78.0; EG: 84.8; CG: 84.8.	Community centres from a non-governmental organisation
Yusupov et al., 2022 [31]	Canada	Mixed methods	Total: 40.	N/A	N/A	Community dwelling

CG: control group; EG: experimental group; RCT: randomised controlled trial; IG: information group; GSG: goal-setting group; GSM: goal-setting with mentoring group; FCP: falls care pathway; PCP: polypharmacy care pathway; LCP: loneliness care pathway; MCP: frailty/medical care pathway.

**Table 3:** Quality assessment of the included studies.

Authors	Study Design	Tool for Assessment	Overall Quality
Beyer et al. [18]	RCT	JBI for RCT	Low
Clare et al. [19]	RCT	JBI for RCT	Medium
Davodi et al. [20]	RCT	JBI for RCT	Low
Franse et al. [21]	QES	JBI for QES	High
Franse et al. [22]	MMS	MMAT	Medium
Green et al. [23]	QS	JBI for QS	Medium
Hsu et al. [24]	QES	JBI for QES	Medium
Lee et al. [25]	RCT	JBI for RCT	Medium
Patzelt et al. [26]	QS	JBI for QS	High
Schwingel et al. [27]	QES	JBI for QES	Low
Uemura et al. [28]	RCT	JBI for RCT	High
Van Dongen et al. [29]	RCT	JBI for RCT	Medium
Wong et al. [30]	RCT	JBI for RCT	High
Yusupov et al. [31]	MMS	MMAT	Medium

## Data Availability

Data are contained within the article and the Supplementary Material.

## Supplementary Materials

The following supporting information can be downloaded at https://www.mdpi.com/article/10.3390/geriatrics10010031/s1, Table S1: Randomized controlled trial studies quality appraisal, Table S2: Quasi-experimental studies quality appraisal, Table S3: Mixed-methods studies quality appraisal, Table S4: Qualitative studies quality appraisal, Table S5: PRISMA checklist. References [14,15,16,17,18,19,20,21,22,23,24,25,26,27,28,29,30,31] are cited in the Supplementary Materials.

## Abbreviations

The following abbreviations are used in this manuscript:

WHO	World Health Organization
PICOS	Patients, Interventions, Comparators, Outcomes, and Study design
MMAT	mixed methods appraisal tool
JBI	Joanna Briggs Institute
MMS	mixed methods study
PA	physical activity
QES	quasi-experimental
QS	qualitative study
RCT	randomised controlled trial
MoCA	Montreal Cognitive Assessment
FCAS	Florida Cognitive Activities Scale
CVLT	California Verbal Learning Test
CED-D 10	German Version of the Center of Epidemiological Studies Depression Scale
GSE	General Self-Efficacy Scale

## Appendix A. Included Studies

**Study**	**Preventive Pathway Characteristics**	**Professional Figures Involved**	**Outcomes**	**Results**
Beyer et al. [18]	Live Active Preventive pathway: self-positive perception of ageing intervention: (1) a quiz on ageing, (2) information on benefits of more positive approach to health, (3) homework presented and discussed in the group, and (4) intervention unit starting with a presentation and discussion of homework. Participants wrote some general or positive personal aspects of ageing. Duration: 12 weeks.	Kinesiologist and psychologists	Self-perceived aging process (SPA): AgeCog Battery, Physical Performance, Short Physical Performance Battery (SPP). Depression symptoms: 15-item German version of the Center of Epidemiological Studies Depression Scale: Mental health. Health-related quality of life: SF-8 health status.	EG showed a significant increase in SPA. Physical Performance: SPPB increased in both EG and CG. Depressive symptoms: significantly decreased in EG vs. CG.
Clare et al. [19]	Agewell preventive pathway: structured goal-setting process based on physical activity, cognitive activity, physical health, nutrition, and social engagement. Duration: 12 months.	Research team	Primary outcomes: physical activity levels, Physical Activity Scale for the Elderly (PASE); cognitive activity, Florida Cognitive Activities Scale (FCAS) and Montreal Cognitive Assessment (MoCA); immediate and delayed recall ability, California Verbal Learning Test (CVLT); executive function, Delis–Kaplan Executive Function System (D-KEFS), trail making, and verbal fluency. Secondary outcomes: General Self-Efficacy Scale (GSES), Center for Epidemiological Studies Depression Scale (CES-D), quality of life, and CASP-19 measure. Physical health: senior fitness test (SFT), Body Mass Index, body fat percentage, cholesterol level, and cardiovascular risk QRISK2. Nutrition: Mediterranean Diet Adherence Screener (MEDAS).	The intervention was feasible and acceptable, with no adverse effects. Physical activity levels increased in GS and GM compared with GC (effect size of 0.37). Cognitive activity increased in GS and GM compared with GC (effect size of 0.15). Memory, executive function, cholesterol levels, aerobic capacity, flexibility, balance, grip strength, and agility increased in GS and GM compared with GC (effect sizes ≥ 0.2). GM obtained more benefits compared with GS (effect sizes ≥ 0.2) in physical activity, body composition, global cognition, and memory but not in other domains.
Davodi et al. [20]	Active Ageing preventive pathway: Group training sessions focused on Nutrition, Physical Activity, Responsibility, Stress management, Communications, and Spiritual aspects. Duration: Three groups of ten people; the educational sessions consisted of six sessions for each group; every week, one training session was held.	Physicians, psychologist, kinesiologist, and religious expert	Active Aging Questionnaire with specific subdomain: active mind maintenance. Physical functional activity, productive engagement, social institutional participation, and social contact agent attitude.	Active ageing: Active mind maintenance increased in EG compared with CG, *p* < 0.05. Physical–functional activity, social contacts, productive engagement, and social institutional participation between the CG and EG after the intervention, *p* < 0.05. Agent attitude, no difference was shown between the CG and EG after the intervention, (*p* = ns).
Franse et al. [21]	Urban Health Centers Approach: (1) Preventive health assessment at home or health centres. (2) Shared decision making. (3) Care Pathway: fall prevention actions, actions addressing polypharmacy (adherence and/or appropriate prescribing); actions addressing loneliness; frailty and other medical actions; frailty actions and other medical care by the healthcare provider.	Care coordinator, nurses, physician, general practitioners, and kinesiologist	Primary general health outcomes: 15-item Tilburg Frailty indicator (TFI), SHARE-Frailty instrument, Short Nutritional Assessment Questionnaire 65+ (SNAQ-65+), 18-item Groningen activity restriction scale, Global Activity Limitation Index (GALI), short-form (SF-12v2), and 5-item mental well-being scale of the SF-36. Secondary specific health outcomes of care pathways: Falls Efficacy Scale International (FES-I), 10 items of the Medication Risk Questionnaire (MRQ-10), and 6-item version of the Jong Gierveld loneliness scale. Care use: the number of visits to a medical doctor, the number of days admitted to a hospital, and the hours per week receiving help in caring for oneself.	Primary general health outcomes: Frailty TFI was lower in EG vs. CG, *p* < 0.05; mental well-being SF-36 was higher EG vs. CG, *p* < 0.05; physical health-related quality of life was better in EG vs. CG, *p* < 0.05. Secondary specific health outcomes of care pathways: The FES-I and loss of independence score were lower, and mental health-related quality of life and mental well-being were higher among EC vs. CG, *p* < 0.05. EG showed less recurrent falls vs. CG, *p* < 0.05. Regarding care use, the number of hours per week needing household help due to health problems was reduced among persons in the intervention group compared with persons in the control group.
Franse et al. [22]	Urban Health Centres approach (UHCE-approach): The Urban Health Centres Europe approach was a preventive, coordinated care approach that aimed to promote healthy ageing with (1) reduction in falls, (2) polypharmacy, and (3) loneliness.	Care coordinator, physician, general practitioners, nurses, informal caregiver, physiotherapists, occupational therapists, exercise experts, psychologists, social workers, pharmacists, and volunteers	Qualitative results: satisfaction with UHCE-approach. Quantitative results: logbook with quantitative information of the delivery and involvement of the older person in the three stages of the UHCE-approach.	Quantitative results: Across all cities, 28.6% enrolled in the falls care pathway, 23.0% enrolled in the loneliness care pathway, 13.7% enrolled in the polypharmacy care pathway, and 9.9% enrolled in the frailty/medical care pathway. Overall, 82.1% of persons in all cities felt that they had benefited from the health assessment, and 85.4% of persons felt that it had been worth the time and effort. Qualitative results: Persons were generally satisfied with the UHCE-approach. Facilitators: The feeling of being cared for improved in older people. The motivation to take action for their health increased. Barriers: Mistrust among older persons towards unfamiliar care professionals and activities, feeling embarrassed, or lacking confidence about engaging in activities. Health constraints of older persons.
Green et al. [23]	Being Your Best preventive pathway: (1) Moving Well: promoting involvement in multicomponent physical activity, through group fitness activities in the community or educational booklets for home-based intervention. (2) Eating Well: Healthy eating booklet, alongside connection with existing community groups revolving around food, such as casserole clubs. (3) Thinking Well: cognitive training through existing community-based group activities, online modules comprising games that improve memory, cognitive abilities, and problem-solving skills or cognitive exercises. (4) Connecting Well: social support based on connecting the participant with an existing volunteer-based conversational phone support service or existing social community groups. Duration: 6 months.	Hospital nurse, general practitioners	Focus groups and interview on the following topics: what frailty means; evidence for effective strategies to support physical function, cognition, social connection, and nutrition; what strategies should be included and what they should be called; accessible community services and support networks that might address one or more aspects of frailty.	Healthcare consumers: What frailty means: All participants described frailty incorporating physical, cognitive, social, and nutritional aspects. Moving well: Regular exercise would help mitigate frailty and facilitate recovery after hospital discharge considering self-motivation. The general practitioner should make referrals to community services or suggest individual interventions. Thinking Well: Cognitive decline is an indicator of frailty and decline in well-being. Attending community groups, using community-based services, and learning-together sessions were also recommended, as were the use of smartphones or computers, playing interactive video games, and engaging with web-based activities. Connecting Well: Most participants agreed that being socially connected was an integral part of their personal well-being. Eating Well: Healthy eating was important for general well-being, but some participants found cooking for themselves tedious and fresh produce expensive. Meal preparation can become boring and tedious, so a food chart including daily nutritious recipes was thought to be helpful. Healthcare professionals: People who are frail or at risk of being frail should engage with physical exercise as a preventive measure for maintaining and improving function. Appropriate nutrition was also considered to be an integral part of mitigating frailty, as was frequent "brain training" for mitigating the effects of cognitive frailty. For some, social connection was important for cognitive stimulation, but all agreed that it was important for social well-being. Co-design results: The co-design process resulted in the refinement of the Being Your Best program to incorporate a holistic approach, addressing four domains supported by research evidence.
Hsu et al. [24]	Preventive pathway: (1) Concept and preparation for successful aging: the lecture introduced the idea of successful aging, risk and protective factors for successful aging, strategies for successful aging, the importance of preparing for old age in life, and the framework of the whole program. (2) Physical activity: the unit introduced content about common physical activities and a fitness examination and then provided a demonstration of easy physical activity exercises that can be performed at home. An elastic band was provided to each participant to use in the physical activity exercise. (3) Nutrition and diet for older people: the educational content included reference material about daily diet, six categories of food and nutrition, portion size and calories, appropriate nutrition suggestions for older adults, and the DASH diet. (4) Chronic disease prevention and management: this unit included the management of common chronic diseases for older adults, prevention strategies, and medication usage knowledge. (5) Emotional health and coping with stress: this unit was designed against the background of cognitive behaviour therapy and introduced self-awareness of emotions and distress, as well as practice on coping skills for more balanced psychological well-being. (6) Cognitive function training. (7) Family relationships. (8) Financial security. (9) Internet use. Duration: 12 weeks.	Expert in gerontology, physical therapist, kinesiologist, attending physician, expert and practitioner in the field of mental health, experts in family relationships, and lawyer with expertise in social protection	Primary outcomes: physical health: self-rated health (scores of 1–5), activities of daily living (IADLs), Nagi scale, and number of chronic diseases; mental health: depressive symptoms (CES-D 10); cognitive function: MoCA (Montreal Cognitive Assessment); stress coping strategies (COPE scale); lifestyle and health literacy; diet behaviours (ad hoc questionnaire for risk); sedentary behaviours (hours per day); regular exercise (number of sessions per week). Other outcomes: social and economic dimension; emotional support and instrumental support from family and friends (ad hoc questionnaire); financial security; internet use; life satisfaction.	General results: Strategies for adapting to old age and reducing ineffective coping were significantly improved in the P2P group. The ability to search for health information improved in the P&D group, and knowledge of finance-related law increased in the P2P group. In the EG P2P group, self-rated health improved, nutrition risk improved, negative coping strategies decreased, and financial security knowledge increased significantly. In EG P&D, diet behaviour and diet literacy increased, the use of the selection adaptation strategy for aging increased, and life satisfaction increased significantly. CG participants increased their self-rated health, their diet behaviour improved, the use of the selection adaptation strategy for aging increased, and the financial preparation percentage increased. Lifestyle: Diet behaviour risk was significantly reduced for the EG P&D group compared with CG, (B = 0.073, *p* < 0.05). Mental health: Cognitive function increased for both the EGP2P and EGP&D groups, although this was not significant. Stress coping strategies: only the selection adaptation strategy significantly increased for both the EGP2P and EGP&D groups. Regarding coping with stress, the only significant effect observed was a reduction in the use of emotion-focused coping for the P2P group. Social and economic dimensions, life satisfaction, and internet use: The family support rating was reduced for the EG P2P group. The support from friends was significantly changed. The EGP&D group significantly increased their ability to search for health information online. Knowledge about financial security law increased in the EGP2P group.
Lee et al. [25]	Preventive pathway: physical activity (45 min of exercise session), nutrition (15 min of nutrition advice), cognitive health (1 h of cognitive training), and homework (participants engaged in homework based on cognitive activities or physical activity tasks). The programme was designed to nurture healthier lifestyles via simple participatory training activities. After three training sessions, the research team gave a lesson on healthy ageing, lifestyle modification, and managing chronic conditions. Duration: 2 h training sessions for 12 months.	Nurses, kinesiologist, physician, nutritionists, and pharmacists	Quantitative outcomes: health-related quality of life (SF-36), cognitive performance, Montreal Cognitive Assessment (MoCA), and Standard Set Older Person metrics (ICHOM). Qualitative outcomes: Trained research nurses also interviewed participants face-to-face at each 3-month follow-up to collect data on demographic characteristics, health behaviours including tobacco or alcohol use during the previous 6 months, and self-reported physician-diagnosed medical conditions. Blood samples.	Live Active Preventive pathway self-positive perception of aging intervention: (1) a quiz on ageing, (2) information on benefits of more positive approach to health, (3) homework was presented and discussed in the group, and (4) intervention unit started with a presentation and discussion of the homework. Participants wrote some general or positive personal aspects of ageing. Duration: 12 weeks.
Patzelt et al. [26]	Reaching the Elderly Preventive Pathway. Preventive home visit. The goal is to prevent or delay the need for long-term care, promote health, and help older people lead an independent and self-determined life.	Preventive health counsellors, social workers, nutritionists, and social scientists	Qualitative outcomes: semi-structured interview of 45 min and focus groups on the following topics: understanding health in old age, experience with preventive measures, and preferred way to be addressed and approached and preferred information channels.	Understanding of health in old age: Women primarily associated health in old age with social participation, personal well-being, and independent living. Men tended to have a more functionally oriented view and associated healthy ageing with physical activity, mobility, and performance. Experience with preventive measures: The utilised services ranged from memory training and cooking classes to holistic approaches like healthy back, pelvic floor exercise, senior citizen dancing, Qigong, and autogenic training. Men tended to associate preventive measures with physical exercise-related services more than women. Exercise classes were mentioned in all focus groups. Swimming and water aerobics were particularly popular. Preferred way to be addressed and approached and preferred information channels: We informed them about the program by phone. Television and radio were not considered to be sources of information about preventive health opportunities. There was hardly any awareness of the internet as a potential source. Both groups relied on their children and grandchildren for information from the internet.
Schwingel A. et al. [27]	Preventive Pathway Abuelas en Acción (AEA) or Grandmothers in Action: physical activity, nutrition, and stress management. The program comprised three core elements: individual meetings, six educational workshops, and weekly follow-up motivational phone calls. Duration: a 6-month active phase and a 3-month maintenance phase.	Trained promotoras and the church/priests	Primary outcomes: nutrition: Food frequency questionnaire (FFQ); depressive symptoms: Spanish translation of the Center for Epidemiological Studies Depression CES-D and Boston 10: Depression Scale; physical activity (PA) levels: Actigraph GT3X plus. Secondary outcomes: Qualitative assessment: individual interviews.	General results: The program was effective in improving participants’ eating behaviours, increasing their physical activity levels, and lowering their depressive symptoms. Promotoras felt motivated and sufficiently prepared to deliver the program. Physical activity levels: The percentage of participants classified as active significantly increased over time. Qualitative results: The interviews indicate how appealing walking is to Latinas and how the program educated and motivated them to walk more. Participants also demonstrated an ardent commitment to being active in general. Furthermore, seven participants mentioned that they had created their own strategies for incorporating physical activity into their daily lives, especially around the house. Nutrition: The number of people that increased consumption of healthy meals increased, *p* < 0.05. The number of days per week in which fruits were consumed increased, *p* < 0.05. There was a reduction in the number of days in which fried food was consumed, *p* < 0.05. Participants indicated that they followed the program’s dietary education guidelines and increased their intake of fibre by eating more fruit, vegetables, and whole grain. Depression: depressive symptoms decreased. *p* < 0.05. Participants mentioned that the program had a positive effect on their mental health and enriched their social lives.
Uemura et al. [28]	Active Learning preventive pathway: The program aimed to promote behavioural changes in daily life and self-management according to the health status of the individual. The themes of this active learning program focused on the role of exercise, diet/nutrition, and cognitive activity to promote the health of older adults. The weekly session was regarded as an opportunity for older adults to learn about and plan strategies to lead a healthy lifestyle. (1) Exploratory learning for homework, (2) discussions as a part of group work in the classroom, and (3) self-planning and the implementation of health promotion strategies in daily life. Duration: 24-week intervention period.	Physical therapists and kinesiologist	Health literacy: Health Literacy Scale (HLS)-14; lifestyle behaviour: accelerometers, food intake frequency of consumption of 10 foods, life-space assessment, the grip strength of the dominant hand was measured by using a portable grip strength dynamometer (T.K.K.5401, Takei Ltd., Niigata, Japan), gait speed was assessed by using the 5 m walking test, 15-item Geriatric Depression Scale, and The 6-item Lubben Social Network Scale.	Health literacy: The EG increased the HLS score, *p* < 0.05. There was a greater increase in critical health literacy scores in the EG than in the CG, but p:ns. Lifestyle behaviour: Step counts increased in EG vs. CG, *p* < 0.05; MVPA increased in EG vs. CG, *p* < 0.05; dietary variety increased in EG vs. CG, *p* < 0.05; frequency of consumption of different foods increased in EG vs. CG, *p* < 0.05; life-space mobility increased in EG vs. CG, *p* < 0.05; social network size increased in EG vs. CG, *p* < 0.05; grip strength increased in EG vs. CG, *p* < 0.05; gait speed increased in EG vs. CG, *p* < 0.05; depression symptomatology increased in EG vs. CG, *p* < 0.05.
van Dongen et al. [29]	The SLIMMER preventive pathway: The standardised SLIMMER intervention was tailored to participants’ individual needs. Dietary intervention: individually tailored dietary advice given in five to eight individual consultations and one group session. The aim was to adopt a sustainable healthy dietary pattern and also reduce body weight by 5–10%. Dietary advice was given by a primary healthcare dietitian. Physical activity intervention: combined aerobic and resistance exercise programme, supervised by a physiotherapist. The aim was to obtain and maintain an active lifestyle, that is, moderate-intensity PA for at least 30 min/d at least five days per week. Case management: practice nurses were appointed as case managers of the intervention programme to enhance participant compliance and the feasibility of implementation. Maintenance programme. Duration: 10 months.	General practitioners, practice nurses, dieticians, physiotherapist, and kinesiologist	Quantitative outcomes: Health outcomes and lifestyle behaviours, fasting insulin, weight, dietary intake, and physical activity. Qualitative outcomes: interviews to evaluate recruitment procedures and barriers procedures, acceptability, adherence, satisfaction, and implementation integrity.	The SLIMMER intervention was well received by both participants and health professionals. A higher dose received and participant acceptance were associated with improved health outcomes and dietary behaviour but not with physical activity behaviour. The intervention programme was implemented as planned and was applicable in Dutch primary healthcare.
Wong et.al [30]	Preventive Pathway Health Social Partnership Program (HSPP): (1) Zoom meeting in order to asses health needs; (2) individualised health education based on the needs: financial support, nutritional advice, physical exercise, and health counselling for the participants; (3) monitoring adherence to the program; (4) final evaluation using telephone calls and meetings.	Registered nurse, community workers, social worker, traditional Chinese medicine practitioner, and group of general practitioners	Primary outcomes: self-efficacy: Chinese version of the General Self-Efficacy Scale; quality of life: 12-item Short Form Health Survey version 2—Chinese (HK) version (SF-12v2HK); healthcare services utilisation (i.e., numbers of visits, visited clinics/centres, and hospital admission). Secondary outcomes: barriers and facilitators, semi-structured group interviews of the centre staff members, managers, providers, and participants.	Self-efficacy: The mean self-efficacy scores increased in the EG and decreased in the CG, *p* < 0.05. Healthcare services utilisation: Both in the EG and CG, the use of health services decreased, except for the number of unplanned general practitioner visits, which decreased sharply in the EG but increased in the CG. Barriers and facilitators: Centre closure was a barrier to program adoption, as they could not receive updates regarding the program, which subsequently led to their non-participation. Significant changes in operation and service delivery, including redeployment of staff, which increased existing workload. Centre staff manager highlighted key issues which served as barriers to implementing new programs and continuing with existing ones. Facilitators: existing relationship and trust between community centre staff and the older adults, promoting the program among staff.
Yusupov et al. [31]	Memory and Aging preventive pathway: an online memory program modelling an empirically validated in-person memory intervention called The Memory & Aging Program. The programme was tailored to older adults’ specific needs.	Researcher team, clinicians, E-learning designers, patient advisors, research assistants, and administrative staff	Qualitative study: Scavenger Hunt measure from TUNSGTEN for each module that assessed usability of new technologies in interactive settings by indicating yes or no. Pilot Study A: online questionnaires and telephone interviews; participants were then emailed a link to register for the online Memory and Aging Program. Once registered, they were able to complete the pre-intervention questionnaires and watch the introduction videos. Pilot Study B: The Memory Knowledge Quiz, The Memory Toolbox, Lifestyle behaviour change, Program-specific goal attainment, and general feedback.	Qualitative study: All participants found at least one of the three modules easy to use. Pilot Studies A and B: Cognitive function showed improvement. A total of 70% of participants reported a lifestyle change after completing the program. They expressed enjoyment of the various presentation formats. The Memory Toolbox questionnaire indicated that all but one participant acquired and applied a new memory strategy.
