# Supplementary material for: Preventive Pathways for Healthy Ageing: A Systematic Literature Review
Source: Geriatrics (Basel). 2025 Feb 18;10(1):31. doi: 10.3390/geriatrics10010031 (PMC11855837; doi:10.3390/geriatrics10010031)
Supplement: Supplementary file 1 [file geriatrics-10-00031-s001.zip › geriatrics-3394831-supplementary.pdf]

Supplementary material

Table S1. Randomized controlled trial studies quality appraisal

Study	1	2	3	4	5	6	7	8	9	10	11	12	13	Overall
Beyer et al., 2019	No	Yes	Unclear	N/A	N/A	Yes	Unclear	Yes	Yes	Unclear	Yes	Yes	Yes	Low
Davodi et al., 2023	Yes	Yes	Yes	N/A	N/A	Yes	Yes	Yes	Yes	Unclear	No	Yes	Yes	Low
Wong et al., 2022	Yes	Yes	Yes	N/A	N/A	Yes	Yes	Yes	Yes	Yes	Yes	Yes	Yes	High
van Dongen et al., 2016	Yes	Yes	Yes	N/A	N/A	Yes	Unclear	Yes	Yes	Unclear	Yes	Yes	Yes	Medium
Lee et al., 2021	Yes	Yes	Yes	N/A	N/A	Yes	Yes	Yes	Yes	Unclear	Yes	Yes	Yes	Medium
Clare et al., 2015	Unclear	Unclear	Yes	N/A	N/A	Yes	Unclear	Yes	Yes	Yes	Yes	Yes	Yes	Medium
Uemura et al., 2021	Yes	Yes	Yes	N/A	N/A	Yes	Yes	Yes	Yes	Yes	Yes	Yes	Yes	High

Questions

1. Was true randomization used for assignment of participants to treatment groups?
2. Was allocation to treatment groups concealed?
3. Were treatment groups similar at the baseline?
4. Were participants blind to treatment assignment?
5. Were those delivering the treatment blind to treatment assignment?
6. Were treatment groups treated identically other than the intervention of interest?
7. Were outcome assessors blind to treatment assignment?
8. Were outcomes measured in the same way for treatment groups?
9. Were outcomes measured in a reliable way?
10. Was follow up complete and if not, were differences between groups in terms of their follow up adequately described and analysed?
11. Were participants analysed in the groups to which they were randomized?
12. Was appropriate statistical analysis used?
13. Was the trial design appropriate and any deviations from the standard RCT design (individual randomization, parallel groups) accounted for in the conduct and analysis of the trial?

Reference Barker TH, Stone JC, Sears K, Klugar M, Tufanaru C, Leonardi-Bee J, Aromataris E, Munn Z. *The revised JBI critical appraisal tool for the assessment of risk of bias for randomized controlled trials.* JBI Evidence Synthesis. 2023;21(3):494-506

Table S2. Quasi-experimental studies quality appraisal

Study	1	2	3	4	5	6	7	8	9	Overall
Franse et al., 2018	Yes	Yes	Yes	Yes	Yes	Yes	Yes	Yes	Yes	High
Schwingel et al., 2017	Yes	N/A	N/A	No	Yes	Unclear	N/A	Yes	Yes	Low
Hsu et al., 2018	Yes	Yes	Yes	Yes	Yes	Unclear	Yes	Yes	Yes	Medium/High

Questions

1. Is it clear in the study what is the ‘cause’ and what is the ‘effect’ (i.e. there is no confusion about which variable comes first)?
2. Were the participants included in any comparisons similar
3. Were the participants included in any comparisons receiving similar treatment/care, other than the exposure or intervention of interest?
4. Was there a control group?
5. Were there multiple measurements of the outcome both pre and post the intervention/exposure?
6. Was follow up complete and if not, were differences between groups in terms of their follow up adequately described and analyzed?
7. Were the outcomes of participants included in any comparisons measured in the same way?
8. Were outcomes measured in a reliable way?
9. Was appropriate statistical analysis used?

Reference Joanna Briggs Institute. *Critical Appraisal Checklist for Quasi-Experimental Studies.*

Table S3. Mixed-methods studies quality appraisal

Study	S1	S2	1.1	1.2	1.3	1.4	1.5	2.1	2.2	2.3	2.4	2.5	3.1	3.2	3.3	3.4	3.5	4.1	4.2	4.3	4.4	4.5	5.1	5.2	5.3	5.4	5.5	Overall
Franse et al., 2019	Yes	Yes	Yes	Yes	Yes	Yes	Yes	Yes	Yes	Yes	Yes	Yes	Yes	Yes	Yes	N/A	Yes	Yes	Yes	Yes	N/A	Yes	Yes	Yes	Yes	Yes	Yes	Moderate
Yusupov et al., 2022	Yes	Yes	Yes	Yes	Yes	Yes	Yes	Yes	Yes	Yes	Yes	Yes	N/A	Yes	Yes	N/A	Yes	Yes	Yes	Yes	N/A	Yes	Yes	Yes	Yes	Yes	Yes	Moderate

Questions

Screening Questions

- S1. Are there clear research questions?
- S2. Do the collected data allow to address the research questions?

1. Qualitative
   - 1.1. Is the qualitative approach appropriate to answer the research question?
   - 1.2. Are the qualitative data collection methods adequate to address the research question?
   - 1.3. Are the findings adequately derived from the data?
   - 1.4. Is the interpretation of results sufficiently substantiated by data?
   - 1.5. Is there coherence between qualitative data sources, collection, analysis, and interpretation?
2. Quantitative randomized controlled trials
   - 2.1. Is randomization appropriately performed?
   - 2.2. Are the groups comparable at baseline?
   - 2.3. Are there complete outcome data?
   - 2.4. Are outcome assessors blinded to the intervention provided?
   - 2.5. Did the participants adhere to the assigned intervention?
3. Quantitative non-randomized
   - 3.1. Are the participants representative of the target population?
   - 3.2. Are measurements appropriate regarding both the outcome and intervention (or exposure)?
   - 3.3. Are there complete outcome data?
   - 3.4. Are the confounders accounted for in the design and analysis?
   - 3.5. During the study period, is the intervention administered (or exposure occurred) as intended?
4. Quantitative descriptive
   - 4.1. Is the sampling strategy relevant to address the research question?
   - 4.2. Is the sample representative of the target population?
   - 4.3. Are the measurements appropriate?
   - 4.4. Is the risk of nonresponse bias low?
   - 4.5. Is the statistical analysis appropriate to answer the research question?
5. Mixed methods
   - 5.1. Is there an adequate rationale for using a mixed methods design to address the research question?
   - 5.2. Are the different components of the study effectively integrated to answer the research question?
   - 5.3. Are the outputs of the integration of qualitative and quantitative components adequately interpreted?
   - 5.4. Are divergences and inconsistencies between quantitative and qualitative results adequately addressed?
   - 5.5. Do the different components of the study adhere to the quality criteria of each tradition of the methods involved?

Reference McGill University. *MMAT - Mixed Methods Appraisal Tool.*

Table S4. Qualitative studies quality appraisal

Study	1	2	3	4	5	6	7	8	9	10	Overall
Patzelt et al., 2016	Yes	Yes	Yes	Yes	Yes	Unclear	Yes	Yes	Yes	Yes	Medium/High
Green et al., 2022	Yes	Yes	Yes	Yes	Yes	Unclear	Yes	Yes	Yes	Yes	Medium/High

Questions

1. Congruity between the stated philosophical perspective and the research methodology
2. Congruity between the research methodology and the research question or objectives
3. Congruity between the research methodology and the methods used to collect data
4. Congruity between the research methodology and the representation and analysis of data
5. There is congruence between the research methodology and the interpretation of results
6. Locating the researcher culturally or theoretically
7. Influence of the researcher on the research, and vice-versa, is addressed
8. Representation of participants and their voices
9. Ethical approval by an appropriate body
10. Relationship of conclusions to analysis, or interpretation of the data

Reference Lockwood C, Munn Z, Porritt K. *Qualitative research synthesis: methodological guidance for systematic reviewers utilizing meta-aggregation.* Int J Evid Based Healthc. 2015;13(3):179–187

Table S5. PRISMA Checklist

Section and Topic	Item	Checklist item	Page
**Title**			
Title	1	Identify the report as a systematic review.	1
**Abstract**			
Abstract	2	See the PRISMA 2020 for Abstracts Checklist.	
**Introduction**			
Rationale	3	Describe the rationale for the review in the context of existing knowledge.	1-2
Objectives	4	Provide an explicit statement of the objective(s) or question(s) the review addresses.	2
**Methods**			
Eligibility Criteria	5	Specify the inclusion and exclusion criteria for the review and how studies were grouped for the syntheses	2-3
Information sources	6	Specify all databases, registers, websites, organisations, reference lists and other sources searched or consulted to identify studies. Specify the date when each source was last searched or consulted.	3
Search strategy	7	Present the full search strategies for all databases, registers and websites, including any filters and limits used.	3
Selection process	8	Specify the methods used to decide whether a study met the inclusion criteria of the review, including how many reviewers screened each record and each report retrieved, whether they worked independently, and if applicable, details of automation tools used in the process.	2-3
Data collection process	9	Specify the methods used to collect data from reports, including how many reviewers collected data from each report, whether they worked independently, any processes for obtaining or confirming data from study investigators, and if applicable, details of automation tools used in the process.	4
Data items	10a	List and define all outcomes for which data were sought. Specify whether all results that were compatible with each outcome domain in each study were sought (e.g. for all measures, time points, analyses), and if not, the methods used to decide which results to collect.	2-4
	10b	List and define all other variables for which data were sought (e.g. participant and intervention characteristics, funding sources). Describe any assumptions made about any missing or unclear information.	2-4
Study risk of bias assessment	11	Specify the methods used to assess risk of bias in the included studies, including details of the tool(s) used, how many reviewers assessed each study and whether they worked independently, and if applicable, details of automation tools used in the process.	4
Effect measures	12	Specify for each outcome the effect measure(s) (e.g. risk ratio, mean difference) used in the synthesis or presentation of results	N/A
Synthesis methods	13a	Describe the processes used to decide which studies were eligible for each synthesis (e.g. tabulating the study intervention characteristics and comparing against the planned groups for each synthesis (item #5)). 4	
	13b	Describe any methods required to prepare the data for presentation or synthesis, such as handling of missing summary statistics, or data conversions.	N/A
	13c	Describe any methods used to tabulate or visually display results of individual studies and syntheses.	4
	13d	Describe any methods used to synthesize results and provide a rationale for the choice(s). If meta-analysis was performed, describe the model(s), method(s) to identify the presence and extent of statistical heterogeneity, and software package(s) used.	N/A
	13e	Describe any methods used to explore possible causes of heterogeneity among study results (e.g. subgroup analysis, meta-regression).	N/A
	13f	Describe any sensitivity analyses conducted to assess robustness of the synthesized results.	N/A
Reporting bias assessment	14	Describe any methods used to assess risk of bias due to missing results in a synthesis (arising from reporting biases).	4-5
Certainty assessment	15	Describe any methods used to assess certainty (or confidence) in the body of evidence for an outcome.	N/A
**Results**			
Study selection	16a	Describe the results of the search and selection process, from the number of records identified in the search to the number of studies included in the review, ideally using a flow diagram.	4-5
	16b	Cite studies that might appear to meet the inclusion criteria, but which were excluded, and explain why they were excluded.	N/A
Study Characteristics	17	Cite each included study and present its characteristics.	6-8
Risk of bias in studies	18	Present assessments of risk of bias for each included study.	5
Results of individual studies	19	For all outcomes, present, for each study: (a) summary statistics for each group (where appropriate) and (b) an effect estimate and its precision (e.g. confidence/credible interval), ideally using structured tables or plots.	N/A
Results of syntheses	20a	For each synthesis, briefly summarise the characteristics and risk of bias among contributing studies	12-22
	20b	Present results of all statistical syntheses conducted. If meta-analysis was done, present for each the summary estimate and its precision (e.g. confidence/credible interval) and measures of statistical heterogeneity. If comparing groups, describe the direction of the effect.	N/A
	20c	Present results of all investigations of possible causes of heterogeneity among study results.	N/A
	20d	Present results of all sensitivity analyses conducted to assess the robustness of the synthesized results.	N/A
Reporting biases	21	Present assessments of risk of bias due to missing results (arising from reporting biases) for each synthesis assessed.	N/A
Certainty of evidence	22	Present assessments of certainty (or confidence) in the body of evidence for each outcome assessed.	N/A
**Discussion**			
Discussion	23a	Provide a general interpretation of the results in the context of other evidence.	8-10
	23b	Discuss any limitations of the evidence included in the review.	10
	23c	Discuss any limitations of the review processes used.	10
	23d	Discuss implications of the results for practice, policy, and future research.	8-10
**Other information**			
Registration and protocol	24a	Provide registration information for the review, including register name and registration number, or state that the review was not registered.	2
	24b	Indicate where the review protocol can be accessed, or state that a protocol was not prepared.	2
	24c	Describe and explain any amendments to information provided at registration or in the protocol.	2
Support	25	Describe sources of financial or non-financial support for the review, and the role of the funders or sponsors in the review.	10
Competing interests	26	Declare any competing interests of review authors.	10
Availability of data, code and other materials	27	Report which of the following are publicly available and where they can be found: template data collection forms; data extracted from included studies; data used for all analyses; analytic code; any other materials used in the review.	10

Section and Topic	Item	Checklist item	Reported
**Title**			
Title	1	Identify the report as a systematic review.	Yes
**Background**			
Objectives	2	Provide an explicit statement of the main objective(s) or question(s) the review addresses.	Yes
**Methods**			
Eligibility criteria	3	Specify the inclusion and exclusion criteria for the review.	Yes
Information sources	4	Specify the information sources (e.g. databases, registers) used to identify studies and the date when each was last searched.	Yes
Risk of bias	5	Specify the methods used to assess risk of bias in the included studies.	Yes
Synthesis of results	6	Specify the methods used to present and synthesise results.	Yes
**Results**			
Included studies	7	Give the total number of included studies and participants and summarise relevant characteristics of studies.	Yes
Synthesis of results	8	Present results for main outcomes, preferably indicating the number of included studies and participants for each. If meta-analysis was done, report the summary estimate and confidence/credible interval. If comparing groups, indicate the direction of the effect (i.e. which group is favoured).	Yes
**Discussion**			
Limitations of evidence	9	Provide a brief summary of the limitations of the evidence included in the review (e.g. study risk of bias, inconsistency and imprecision).	Yes
Interpretation	10	Provide a general interpretation of the results and important implications.	Yes
**Other**			
Funding	11	Specify the primary source of funding for the review.	Yes
Registration	12	Provide the register name and registration number.	Yes
